# Elevated central venous pressure in septic patients is associated with impairment of microcirculatory blood flow

**DOI:** 10.1186/cc10807

**Published:** 2012-03-20

**Authors:** N Vellinga, C Ince, EC Boerma

**Affiliations:** 1 Medisch Centrum Leeuwarden, the Netherlands; 2 Erasmus Medical Center, Rotterdam, the Netherlands

## Introduction

The microcirculation plays a pivotal role in oxygen delivery to the tissue. Microcirculatory alterations have been observed to occur independently of the major inflow variable for microcirculation: mean arterial pressure. According to physiological theory, the microcirculation is considered to be a low-pressure compartment. Maximum optimal central venous pressure (CVP) according to Surviving Sepsis Campaign (SSC) guidelines is 12 to 15 mmHg in mechanically ventilated patients. We hypothesized that a CVP >12 mmHg would hamper microcirculatory perfusion but not diffusion, by acting as outflow obstruction.

## Methods

We retrospectively analyzed combined measurements of CVP and sidestream dark-field derived sublingual microcirculatory variables in patients with severe sepsis or septic shock. Measurements were made 0, 0.5, 2, 12 and 24 hours after resuscitation in accordance with SSC guidelines. Differences in small vessel microvascular flow index (MFI) and total vessel density (TVD) between two groups (CVP ≤12 mmHg and CVP >12 mmHg) were analyzed with a Mann-Whitney U test.

## Results

**Figure 1:**
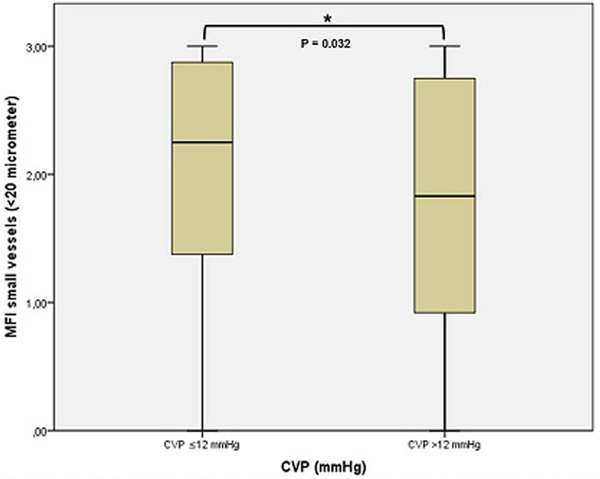
**Boxplots of microvascular flow index (MFI) in patients with a central venous pressure (CVP) ≤12 mmHg or >12 mmHg**.

A total of 345 measurements in 70 patients (APACHE II 21 (6.5) (mean (SD))) were included. MFI in patients with CVP >12 mmHg was significantly lower than in CVP ≤12 mmHg (1.83 (0.92 to 2.75) vs. 2.25 (1.35 to 2.90) (median (IQR)), *P *= 0.032), whereas TVD in both groups did not differ significantly (14 (12.84 to 15.75) vs. 14.3 (13 to 15.8) mm/mm^2^, *P *= 0.38). See also Figure 1.

## Conclusion

In septic patients with CVP >12 mmHg after resuscitation, microcirculatory flow was significantly lower as compared to patients with CVP ≤12 mmHg, whereas capillary density did not differ between groups.

